# Wild chimpanzee behavior suggests that a savanna-mosaic habitat did not support the emergence of hominin terrestrial bipedalism

**DOI:** 10.1126/sciadv.add9752

**Published:** 2022-12-14

**Authors:** Rhianna C. Drummond-Clarke, Tracy L. Kivell, Lauren Sarringhaus, Fiona A. Stewart, Tatyana Humle, Alex K. Piel

**Affiliations:** ^1^ School of Anthropology and Conservation, University of Kent, Canterbury, UK.; ^2^ Centre for the Exploration of the Deep Human Journey, University of the Witwatersrand, Johannesburg, South Africa.; ^3^ Department of Evolutionary Anthropology, Duke University, Durham, NC, USA.; ^4^ Department of Anthropology, University of Michigan, Ann Arbor, MI, USA.; ^5^ Department of Anthropology, University College London, London, UK.; ^6^ School of Biological and Environmental Sciences, Liverpool John Moores University, Liverpool, UK.

## Abstract

Bipedalism, a defining feature of the human lineage, is thought to have evolved as forests retreated in the late Miocene-Pliocene. Chimpanzees living in analogous habitats to early hominins offer a unique opportunity to investigate the ecological drivers of bipedalism that cannot be addressed via the fossil record alone. We investigated positional behavior and terrestriality in a savanna-mosaic community of chimpanzees (*Pan troglodytes schweinfurthii*) in the Issa Valley, Tanzania as the first test in a living ape of the hypothesis that wooded, savanna habitats were a catalyst for terrestrial bipedalism. Contrary to widely accepted hypotheses of increased terrestriality selecting for habitual bipedalism, results indicate that trees remained an essential component of the hominin adaptive niche, with bipedalism evolving in an arboreal context, likely driven by foraging strategy.

## Teaser

Savanna chimpanzee locomotor and postural behavior supports early hominin bipedalism evolving in the trees, not on the ground.

## INTRODUCTION

Obligatory terrestrial bipedalism is a defining feature of modern humans, and its morphological adaptations are critical to distinguishing fossils that fall within the human clade (hominins) from those of other apes (hominoids) over the past 7 million years (Ma) (*1*). The shift to more arid and open environments in the late Miocene-Pliocene (ca. 10 to 2.5 Ma) has played a central role in hypotheses about hominin evolution (*2*, *3*). In particular, the emergence and evolution of bipedalism is often considered to be a key adaptation to more open, dry habitats [termed “savanna,” which includes wooded habitats with a grassy understory rather than only treeless grassland assumed in traditional “savanna hypotheses”; reviewed in (*4*)], in which hominins reduced the time spent in trees and increased terrestrial foraging and traveling as forests retreated (*5*–*8*). Paleoenvironmental reconstructions indicate that early hominins were not living in tropical forests common to most extant apes today (*2*, *3*). Instead, the earliest (putative) fossil hominins, including *Orrorin* (6 Ma) (*9*), *Ardipithecus* (5.8 to 4.4 Ma) (*10*, *11*), and early *Australopithecus* (4.2 to 2.9 Ma) (*12*, *13*), would have moved and foraged in mosaic savanna habitats dominated by woodland with strips of riparian forest vegetation, often termed “savanna-woodland” or “savanna-mosaic” (used hereafter). Compared to tropical forest, these savanna-mosaic habitats would have elicited different selective pressures associated with reduced tree density and increased seasonality (*14*, *15*). For example, savanna-mosaics have temporally and spatially sporadic food sources, as well as greater predatory pressure, which are hypothesized to have selected for bipedalism as an efficient mode of terrestrial travel (*6*, *16*, *17*).

Despite the suggested link between increased terrestriality and the appearance of bipedal adaptations, various lines of evidence support a strong arboreal component in hominin ecology. Fossil hominins show morphological features considered advantageous for arboreal locomotion, such as long upper limbs, mobile shoulder, elbow and wrist joints, and curved phalanges (*15*, *18*). Some or all of these arboreal adaptations are found not only in early hominins [e.g., *Sahelanthropus* (*19*), *Orrorin* (*9*), *Ardipithecus* (*10*, *20*), and *Australopithecus afarensis* (*21*)] but also in later Plio-Pleistocene hominins [e.g., *Australopithecus sediba* (*22*), *Homo naledi* (*23*), and *Homo floresiensis* (*24*)], suggesting that these features were being positively selected, rather than just neutral retentions, and fueling decades of debate around their functional significance in supposedly terrestrial hominin taxa (*15*, *18*). Moreover, isotopic analyses have revealed diversity in early hominin diets in a savanna-mosaic habitat, with some taxa retaining a high C3 component similar to modern savanna chimpanzees, indicative of an arboreal foraging strategy (*25*–*27*). A fundamental question of early hominin evolution remains whether a savanna-mosaic environment acted as a selective driver of terrestriality and thus locomotor bipedalism or, alternatively, whether bipedal locomotion evolved as an arboreal adaptation (e.g., for foraging) that was then exapted for moving terrestrially during later periods of hominin evolution.

**Fig. 1.**
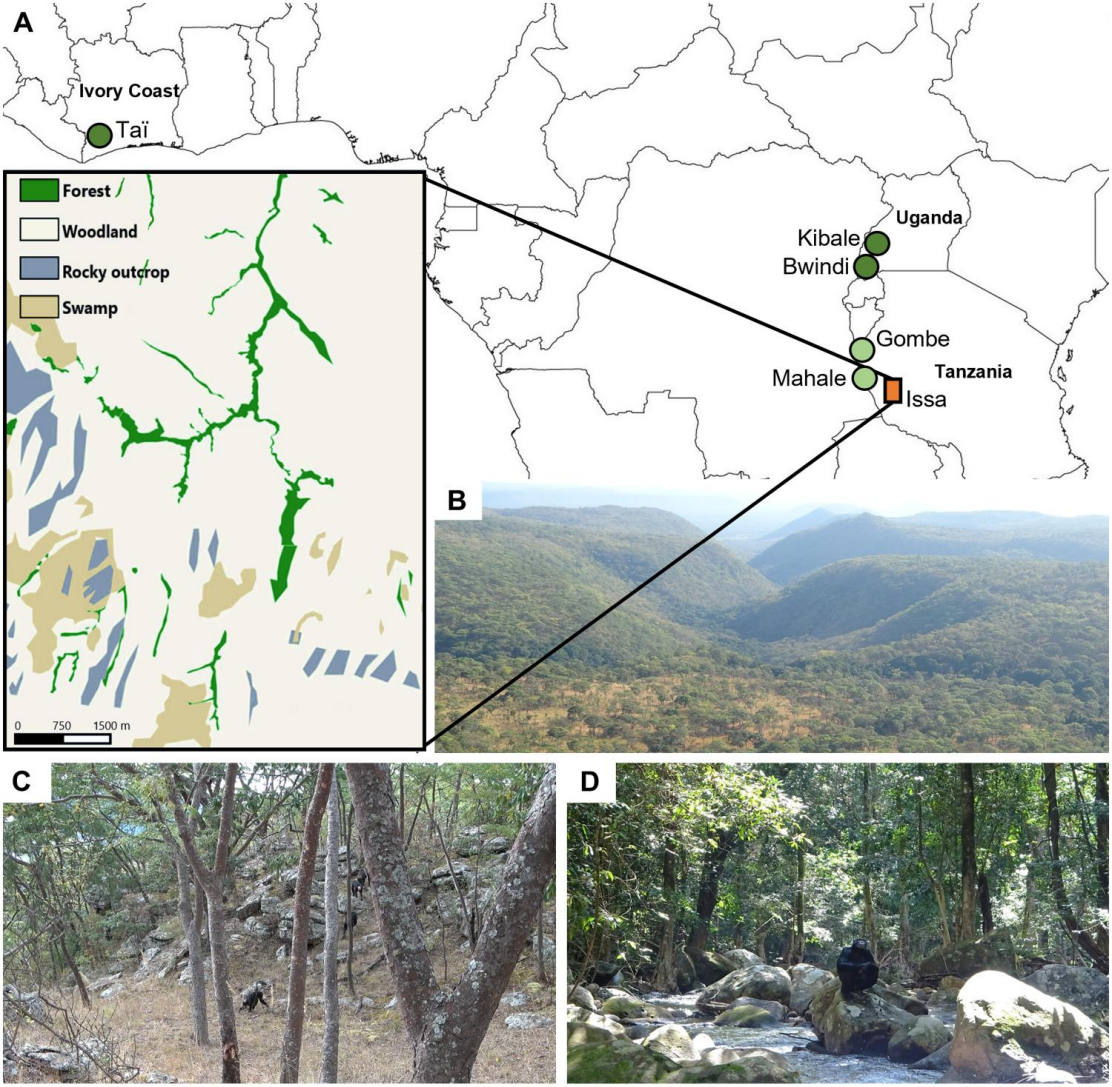
Issa Valley and other chimpanzee study site locations and habitats. (**A**) Issa Valley’s location in western Tanzania relative to Taï (North Group, Ivory Coast), Kibale (Ngogo, Uganda), Bwindi (Uganda), Mahale (M-Group, Tanzania), and Gombe (Kasekela, Tanzania). For comparative purposes, sites are grouped into three categories reflecting the percent forest cover and dryness [following (*35*)]: dense forest (dark green circles; Taï, Kibale, and Bwindi), forest-mosaic (light green circles; Mahale and Gombe), and savanna (orange rectangle; Issa). Forest sites are considered as closed and savanna as open habitat. The Issa study area is a savanna-mosaic habitat with a long dry season that is dominated by miombo woodland, represented in highlighted habitat map and view of site (**B**). Issa’s deciduous miombo woodland (**C**) is classed as open vegetation [grassy understory, broken canopy, low tree density (0.02 trees per square meter), and majority of trees <15 m high], while the evergreen riparian forest (**D**) is classed as closed vegetation, with vine-dense understory, twice the tree density, taller trees, and a more connected canopy than the woodland (table S4). Bwindi is only included in the intersite comparison of bipedal behaviors as no positional behavior frequency data were collected at this site (*38*). Photo credit: R.C.D.-C.

In the absence of direct fossil evidence, and due to difficulties in reconstructing the relationship between behavior and habitat from morphology alone, quantitative studies of locomotor ecology of wild, extant primates have been key to providing valuable insights into how and why bipedalism may have evolved [e.g., (*5*, *28*–*30*)]. Notably, extant apes living in savanna-mosaic habitats analogous to those of early hominins provide ideal models to test the “savanna effect” on ape and, by extension, hominin behavior (*14*, *31*). As our closest living relatives, chimpanzees (*Pan troglodytes*) and bonobos (*Pan paniscus*) are informative analogs (*32*), regardless of whether our last common ancestor was *Pan*-like (*33*, *34*). In particular, chimpanzee habitat range spans the forest-savanna continuum (*35*), and thus, they offer a valuable opportunity to investigate how a large-bodied, semi-arboreal ape adapts its positional (postural and locomotor) behavior and terrestriality to savanna habitats. However, to date, locomotor studies have focused only on forest-dwelling chimpanzees (Fig. 1) (*36*–*38*), overlooking critical comparative data about how behaviors, including bipedalism and degree of terrestriality, vary across habitats.

Issa Valley in western Tanzania is characterized as a savanna-mosaic (*14*, *35*, *39*) similar to the paleoenvironments reconstructed for the early hominins *Orrorin*, *Ardipithecus ramidus*, and *Australopithecus afarensis* (*9*–*13*) and hosts a recently habituated (2018) chimpanzee community (*P. t. schweinfurthii*). The area is a mosaic of miombo woodland with strips of evergreen riparian forest (classed as open and closed vegetation, respectively; Fig. 1). Thus, Issa chimpanzees are well situated for testing the savanna effect on chimpanzee positional behavior, not only through comparison to forest-dwelling communities but also by comparing how individuals adjust their positional behavior across vegetation types within a savanna-mosaic habitat. We quantified locomotor and postural behaviors (table S1) in Issa chimpanzees for 15 consecutive months within the open miombo woodland and closed riparian forest to characterize chimpanzee positional behavior in a savanna-mosaic habitat. Combined with further comparison to chimpanzees living in forest habitats (facilitated by similar behavioral data collection methods across studies), including at Taï (*36*, *40*), Kibale (*37*), Bwindi (*38*), Mahale, and Gombe (*36*, *41*) (Fig. 1) , we test the hypothesis that an open habitat will induce greater terrestriality and terrestrial bipedalism. Our findings offer a unique opportunity to examine whether these positional behavioral changes offer support to the hypothesis that a shift from forest to a more open, savanna-mosaic habitat in the late Miocene-Pliocene was a catalyst for the emergence and evolution of bipedalism in early hominins.

## RESULTS

### Terrestriality in a savanna-mosaic habitat

**Fig. 2.**
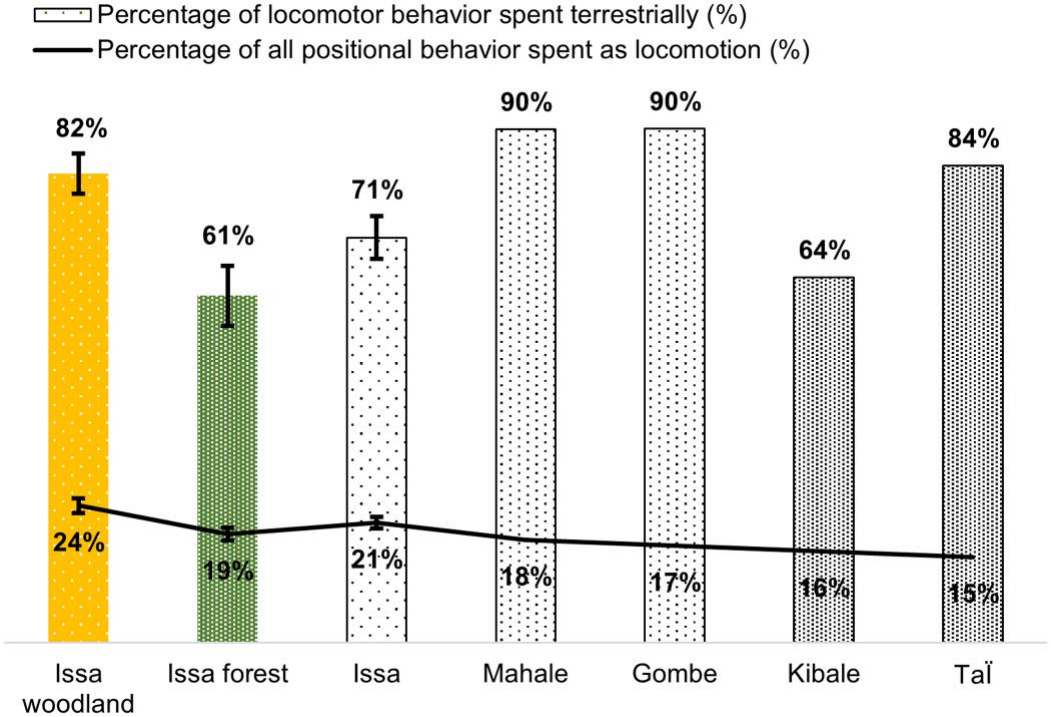
Percentage of time spent in locomotion and, specifically, terrestrial locomotion of Issa Valley chimpanzees in comparison to other chimpanzee communities. The black line represents the percentage of time spent engaged in locomotion out of total positional behavior time (i.e., posture + locomotion = 100%). The bars represent the percentage of only locomotor time that is spent terrestrially. The two vegetation types at Issa Valley are shown independently (woodland as yellow, forest as green) and combined (black and white "Issa" bar) in comparison to the other communities (*36*, *37*, *41*). Dot density represents relative vegetation openness (see Fig. 1 caption for detail). Issa chimpanzees spent significantly more time engaging in locomotion in the woodland versus forest (*t* = −2.69, *P* = 0.02), and across communities, percentage of time spent locomoting decreases with increased forest cover. As a percentage of just locomotor time, Issa chimpanzees spent significantly more time engaging in terrestrial locomotion in the woodland compared to the forest (*t* = 2.834, *P* = 0.02); however, the between-community comparison showed no increase in terrestrial locomotion as forest cover decreases; Issa chimpanzees spent less time locomoting terrestrially than all forest sites except Kibale (see main text for details). Error bars represent SE, available for Issa data only.

We obtained 13,743 instantaneous observations of positional bouts from 13 adults (6 females and 7 males), including a total of 2847 observations of locomotor bouts (table S2). We incorporated data on substrate (i.e., ground versus tree), vegetation type/openness (i.e., woodland versus forest), and contextual activity (e.g., feeding, resting, and traveling; see table S3 for definitions and table S4 for Issa vegetation data). We ran paired *t* tests to assess individual frequencies of positional behavior and terrestriality in contrasting vegetation types (sexes pooled, see table S5 for females and males separately) and Wilcoxon signed-rank tests for analysis of bipedal observations. We found that in open vegetation, Issa chimpanzees spent more time engaging in locomotion (*t* = −2.69, *P* = 0.02) and were significantly more terrestrial (*t* = 2.83, *P* = 0.02) than when in closed vegetation, in particular increasing ground use during locomotion (*t* = 5.99, *P* < 0.001; Fig. 2). We then ran a generalized linear mixed model (GLMM) to investigate whether vegetation type influenced ground use during locomotion while accounting for synergistic interactions with season, activity, and sex, as well as individual variation (table S6). Activity, vegetation type, and season had a significant effect on locomotor terrestriality, whereas sex was only significant during travel but not while foraging (table S6).

We then compared Issa behavioral frequencies to published data from chimpanzees living in forest habitats (*33*, *34*). For comparative purposes, forest sites were considered as closed while savanna-mosaic were considered as open habitat based on dominant vegetation type (see Materials and Methods). As expected, chimpanzees spent more time engaging in locomotion when in an open habitat (Fig. 2). However, contrary to expectation, the proportion of locomotor time spent terrestrially did not increase with habitat openness. Issa chimpanzees spent less time locomoting on the ground than Taï, Mahale, and Gombe chimpanzees (Fig. 2). Issa chimpanzee terrestriality, as well as locomotor behavior (fig. S1), most closely resembled that of densely forested Kibale (Fig. 2).

### Bipedalism

**Fig. 3.**
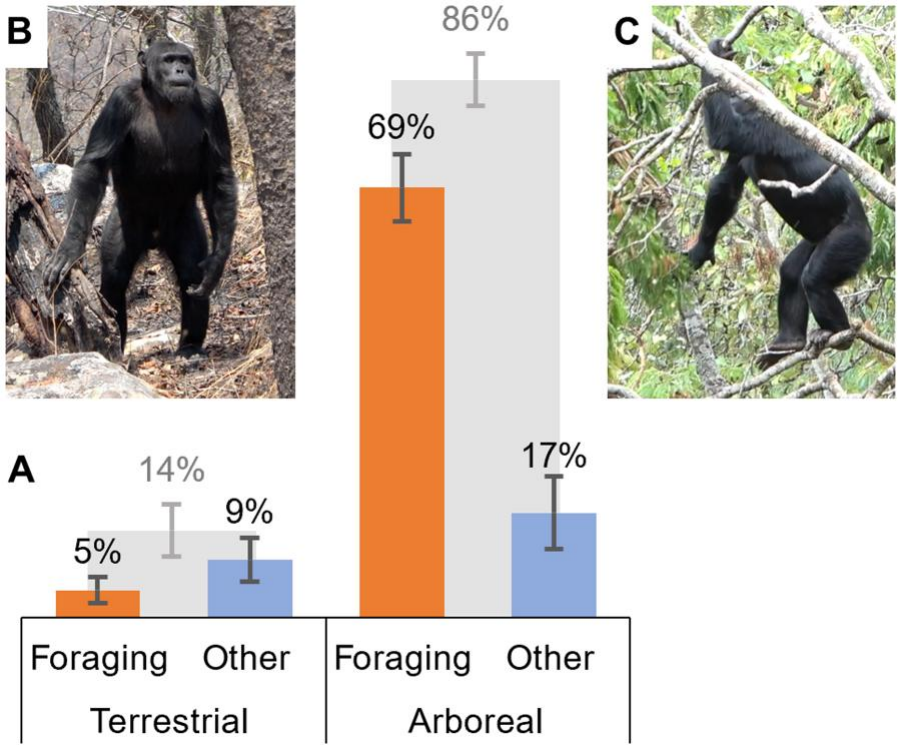
Chimpanzee bipedalism at Issa Valley. (**A**) Percentage of bipedalism (posture and locomotion combined) on terrestrial versus arboreal substrates, depicted by light gray bars behind (summed to 100%). Colored bars in front (summed to 100%) depict percentage of bipedalism on each substrate split across the behavioral context in which the chimpanzees were using bipedalism, divided in foraging (orange) versus other behaviors (blue), i.e., travel, vigilance, and play. Error bars represent SE. Bipedalism in Issa chimpanzees was primarily an arboreal (86%; arboreal versus terrestrial: Wilcoxon *V* = 91, *P* = 0.002) and foraging behavior (73%; forage versus other behavior in arboreal context: Wilcoxon *V* = 170, *P* < 0.001). (**B**) Example of terrestrial (postural) bipedalism. (**C**) Example of arboreal (locomotor) bipedalism during foraging. Photo credit: R.C.D.-C.

**Fig. 4.**
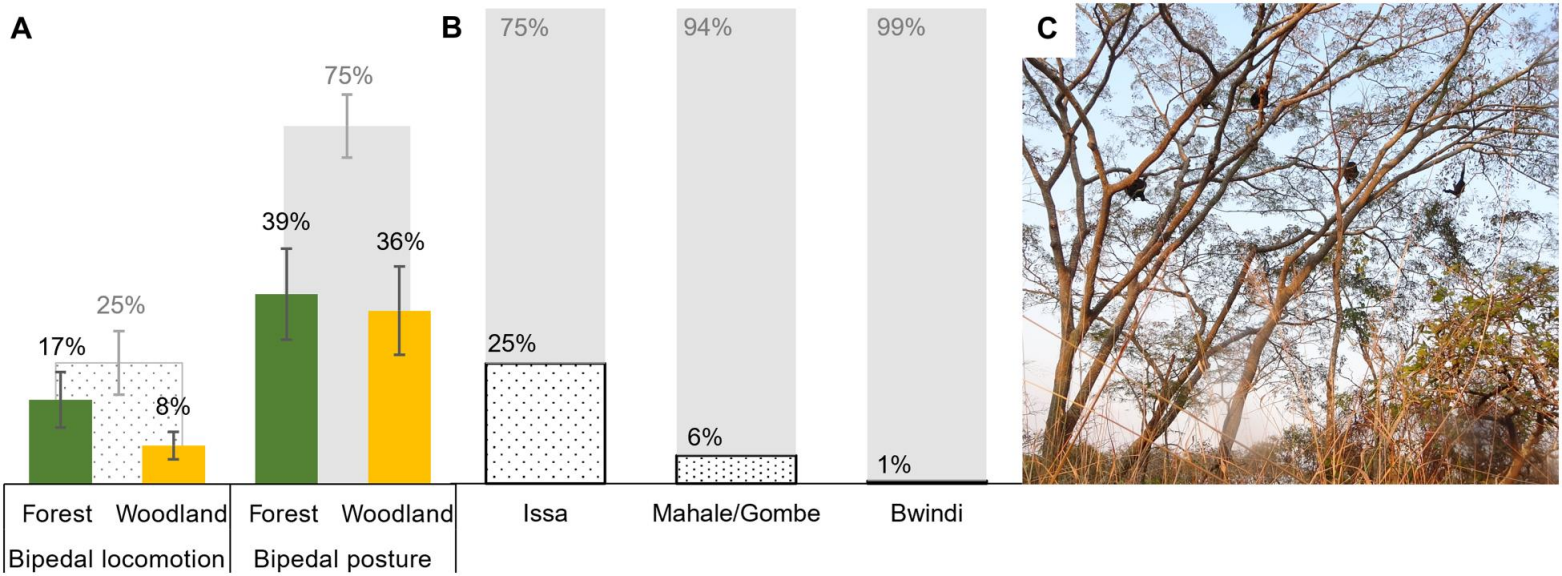
Chimpanzee bipedal locomotion versus bipedal posture. (**A**) Bipedal behaviors at Issa, showing overall percentage of bipedal posture (solid gray) versus bipedal locomotion (dotted; summed to 100%), with each broken down to show use of bipedalism in the forest (green) versus woodland (yellow). Bipedalism was mainly a postural behavior at Issa (75% of all bipedal observations). Although postural bipedalism does not differ in use between vegetation types, there was a trend toward more bipedal locomotion in the forest. Error bars show SE. (**B**) Percentage of total bipedal observations as locomotion (dotted) versus posture (solid gray; summed to 100%) at each chimpanzee site with data available (*37*, *38*, *42*). Issa has the highest percentage of bipedal behavior as locomotion, but there appears to be no relationship between the overall frequency of bipedalism and habitat type. Taï and Kibale are not included as no or insufficient data on the percentage of bipedal behavior spent as locomotion were available (see table S7). (**C**) Example of Issa chimpanzees foraging in open canopy vegetation, in a large tree (>10 m high) with a wide crown and many terminal branch foods (*Brachystegia microphylla*), here hypothesized to select for arboreal bipedalism at Issa. Photo credit: R.C.D.-C.

Bipedalism (postural and locomotor) at Issa occurred primarily on arboreal substrates (as opposed to terrestrial, *V* = 91, *P* = 0.002; 86% of all bipedal observations) and, moreover, primarily during a foraging context (as opposed to other behaviors, *V* = 170, *P* < 0.001; 73% of all bipedal observations; Fig. 3). We observed more bipedality when Issa chimpanzees were in closed compared to open vegetation. While postural bipedal time was the same in both vegetation types (0.77% of total postural time in each vegetation type), Issa chimpanzees used more bipedal locomotion in closed (1.7% of total locomotor time) compared to open vegetation (0.5%; Fig. 4A). However, this locomotor difference was not significant (*V* = 45, *P* = 0.08) potentially due to the rarity of bipedal behavior within their overall positional repertoire (statistical test power = 0.3; tables S5 and S7).

In keeping with values reported from forest chimpanzee communities, bipedalism remained a rare (<1% of all positional behaviors) and primarily arboreal (>80%) behavior in the Issa community (table S7). However, despite no apparent effect of habitat (or vegetation) type on overall frequency or terrestriality of bipedalism, bipedal locomotion was four times more frequent at Issa (25%) than at Mahale and Gombe (6%) (*42*), and even more so compared with Bwindi chimpanzees (0.6%; Fig. 4B) (*38*). Kibale could not be included in the bipedal comparison due to an insufficient sample size (*N* observation hours <100), and data were not available for Taï (table S7).

## DISCUSSION

Our investigation of how Issa chimpanzee positional behavior and terrestriality vary within their savanna-mosaic habitat provides the first test in a living ape of the hypothesis that arid and open environments of the late Miocene-Pliocene acted as a catalyst for hominin terrestrial bipedalism. Variation in Issa chimpanzee positional behavior indicates that terrestriality and bipedalism do not increase within more open habitats and instead offers support for hominin bipedalism evolving within an arboreal context.

### Chimpanzee terrestriality in a savanna-mosaic habitat

Issa chimpanzees increased their terrestriality overall in open (woodland) vegetation compared to the forest, reflecting the importance of vegetation structure on ape substrate use during locomotion (e.g., lower tree density and canopy connectivity in open vegetation reduce horizontal pathways within the canopy, increasing terrestrial travel between feeding trees) (*43*). However, Issa chimpanzees were no more terrestrial in woodland vegetation than chimpanzees living in forest habitats (Fig. 2), suggesting that it is not a simple rule of less trees means more time on the ground. While intersite variation in, e.g., observation technique, level of habituation, and predator presence may exist, contextual activity was an important variable determining chimpanzee ground use during locomotion between open and closed vegetation at Issa (table S6). Following previous studies that demonstrate the importance of spatial and temporal cognition in chimpanzee foraging strategy (*44*–*46*), the lower-than-expected woodland terrestriality at Issa could be explained, in part, by chimpanzees adapting their foraging strategy to remain in the same feeding tree for long periods of time in response to abundant, yet spatially restricted, food sources. Although more data are needed on detailed aspects of tree crown shape, foraging strategy, and traveling patterns to address this hypothesis, dry season feeding at Issa is dominated by *Parinari* and *Brachystegia* sp. (*39*, *47*), both woodland genera characterized by wide canopies and abundant terminal branch fruits (e.g., Fig. 4C). Simultaneously, a foraging strategy of feeding longer in one tree minimizes the use of energetically costly forms of locomotion such as vertical climbing and terrestrial knuckle-walking that would otherwise increase with more terrestrial travel between food patches (*48*). Issa chimpanzee positional behavior is characterized by low frequencies of climbing and knuckle-walking, in combination with a high frequency of suspensory locomotion (a horizontal, terminal branch behavior) compared with other chimpanzee communities (figs. S1 and S2). Combined with Issa also being home to several large, terrestrial predator species with observed chimpanzee encounters, including African wild dogs (*49*), leopards (personal observation), and humans with (domestic) dogs (*50*), arborealism could be positively selected to reduce predation risk (*51*) as well as energy expenditure (*48*).

Differences in the landscape itself may also affect the frequency of terrestriality. Issa, like many early hominin sites (*52*), is defined by steep and rocky terrain (Fig. 1, B and C), and while chimpanzees were observed scaling rocky outcrops, preliminary observations suggest that they circumvent difficult terrain using arboreal routes when possible. Thus, higher selectivity of feeding trees that permit long foraging bouts when in an open habitat, in combination with avoiding challenging terrain, may together reduce terrestrial travel time.

### The hominin arboreal niche

Our results challenge the long-held association between increased terrestriality and the evolution of locomotor bipedalism in early hominins (*5*–*8*, *20*). Whereas previous hypotheses founded on observations of wild chimpanzees have indeed acknowledged the role of arboreal feeding as a driver of bipedal posture, they posit bipedal locomotion evolving as a terrestrial behavior in a more open habitat (*5*, *8*, *38*). Issa chimpanzees remained highly arboreal and did not use more bipedalism in open vegetation (Figs. 3 and 4A). Instead, they used more (arboreal) locomotor bipedalism than forest-dwelling chimpanzees (Fig. 4B), lending support to bipedal locomotion emerging and evolving as an arboreal adaptation in early hominins (*30*, *53*). Combined with the fact that bipedalism was predominantly used while foraging on terminal branches at Issa (Fig. 3A), we further suggest that highly productive, wide-canopy feeding trees favor arboreal (locomotor) bipedalism to safely navigate flexible terminal branches to reach foods and to remain safe from terrestrial predators in an open habitat. This hypothesis is also consistent with the use of bipedal locomotion by orangutans on flexible branches (*30*).

Hominin arboreality is consistent with dental microwear and food mechanical properties of hominins before 4 Ma, showing a C3-rich diet that is similar to that of extant savanna-mosaic chimpanzees (*25*, *54*). Issa chimpanzee positional behavior therefore provides a model for how early hominins could have maintained a C3-rich diet in a savanna-mosaic habitat, foraging arboreally (at equal if not more frequent rates to forest conspecifics) to effectively harvest abundant, but spatially restricted, food sources and to counterbalance energy lost through increased travel distances between widely distributed food patches.

The expansion of more open and arid habitats is thought to have been a catalyst for many changes in hominin behavior, anatomy, and/or physiology over the past 10 Ma (*3*, *55*–*58*). This includes the emergence of hominin bipedalism in the late Miocene-Pliocene as fossils of the earliest (putative) hominins, and multiple australopith taxa are found within savanna-mosaic, rather than closed forest, paleohabitats (*9*–*13*). What remains unclear, however, is what type of selective pressure acted on hominins because of this transition into open habitats since contrasting signals of terrestriality and arboreality leave much uncertainty as to how exactly hominins used these habitats (*15*, *18*). In other words, the simple presence of hominins does not tell us how they were interacting within their paleohabitats. In addition, biomechanical models [e.g., (*59*–*61*)] and internal bone structure [e.g., (*62*–*64*)] highlight greater variation in hominin positional repertoires than previously appreciated. A better understanding of how chimpanzees (and other primates) interact with and alter their behavior in relation to habitat can provide important insights into how and why hominin bipedalism may have evolved. The open, mosaic nature of the Issa Valley means its chimpanzees provide a valuable natural experiment to document how large-bodied, semi-arboreal apes adapt their positional behavior to diverse ecological opportunities and constraints, which, in turn, allows us to test hypotheses about the potential selective pressures that acted on early hominins that lived in analogous savanna-mosaic habitats. We suggest that ecological heterogeneity provided (i) important foods across an increasingly seasonal environment (*39*, *47*) and (ii) the selective pressures that promote and explain the presence, and persistence, of hominin postcranial functional morphology advantageous for bipedal and arboreal locomotion [e.g., (*10*, *15*)]. The high frequency of arboreal behavior in open vegetation, combined with the high frequency of bipedal locomotion at Issa, provides insight into two long-standing debates in paleoanthropology: (i) that bipedalism evolved as an arboreal locomotor behavior before being exapted to a terrestrial context (*30*, *38*, *53*) and (ii) that the arboreal features retained in many early (*9*, *10*, *21*) and even late (*23*) hominins living in open habitats were functionally significant and adaptive. Life in the trees was likely an essential component of the hominin adaptive niche, even as forests retreated (*2*, *65*).

## MATERIALS AND METHODS

### Study site and subjects

During a 15-month study of wild chimpanzees in the Issa Valley, west Tanzania, we obtained 13,743 instantaneous observations of locomotor (2847) and postural (10,896) bouts from 13 adults (table S2). Observations were collected every 2 min during 1-hour focal follows, including information on contextual activity, support, and vegetation type (table S3). Data were collected by R.C.D.-C. and a trained field assistant (trained in data collection and interobserver reliability checked by R.C.D.-C. during 1 month before starting data collection). Data collection methods were similar to previous studies of chimpanzee positional behavior [i.e., instantaneous focal sampling (*66*)] to allow comparisons to other habituated chimpanzee communities (*37*, *40*, *41*). This work was approved by the Tanzania Wildlife Research Institute (TAWIRI) and the Tanzania Commission for Science and Technology (COSTECH), and adheres to guidelines laid out by the International Union for Conservation of Nature (IUCN) Primate Specialist Group Section for Human-Primate Interactions, as well as the American Society of Primatologists’ principles for ethical treatment of nonhuman primates.

### Classification of habitats and vegetation types

To investigate the influence of habitat openness between study sites, chimpanzee sites were classified as dense forest, mosaic forest, or savanna following van Leeuwen *et al.* (*35*). Dense forest is linked to highest forest cover, and therefore tree density, and considered the most “closed,” and savanna the lowest tree density and thus most “open,” with mosaic forest intermediate between the two. Taï, Kibale, and Bwindi were classified as dense forest, Gombe and Mahale National Parks as mosaic forest, and Issa as savanna (Fig. 1) (*35*). Across the savanna-mosaic habitat of Issa, the chimpanzees use predominantly two vegetation types: riparian forest and miombo woodland. The miombo woodland has half the tree density compared with that of the forest such that each habitat at Issa can be considered closed (forest) and open (woodland; Fig. 1 and table S4). In addition, these vegetation types contrast significantly in structural features related to arboreal substrate availability: Tree height, crown height, and canopy cover/connectivity are all greater within the forest compared with the woodland (table S4) (*67*). Moreover, the understory structure in the forest is dense with lianas compared with open and grassy in the woodland (Fig. 1 and table S4). This more detailed structural profile of open and closed vegetation types at Issa highlights vegetation features, apart from tree density, that may influence vegetation “openness” by changing availability of arboreal substrate and chimpanzee terrestriality.

### Positional mode classification

Chimpanzee positional behaviors (locomotor and postural) were defined following the classification scheme set out by Hunt *et al.* (*68*) and reflecting modifications from Sarringhaus *et al.* (*37*) and Thorpe and Crompton (*69*) (table S1). Locomotion was defined as behavior where the focal individual’s center of gravity was displaced from one place to another, and posture as any behavior where the center was not displaced. Bipedalism was defined as any posture or locomotion when the torso was orthograde with weight born primarily on the hindlimbs, the knees and hips were semiflexed to extended, and with minimal contribution from the forelimbs. Monopedal stand was also included with bipedal stand following Thorpe and Crompton (*69*).

### Statistical analysis

To investigate the difference in positional behavior between vegetation types at Issa, observation sessions for each individual were aggregated to single data points within analytical categories to ensure no single individual skewed the results. Interdependence of sequential observations separated by a small-time interval is problematic in the analysis of positional behavior data (*37*, *41*, *70*). However, wild chimpanzee locomotor bouts (defined as the beginning of locomotor activity to stopping locomotion) are regularly interrupted by bouts of rest or change of activity. Combined with locomotion accounting for less than 25% of all scans, it was decided that dependence between locomotor data points was negligible, and all observations of locomotion were analyzed. Postural observations were not included in analysis of substrate use to avoid aforementioned problems of interdependence, and as postural behavior was not the focus of this study. For investigating frequency and use of bipedalism, postural bouts were included with locomotor bouts for analyses unless stated otherwise. However, interdependence was not considered a problem because the rarity and short duration of bipedal behavior meant that sequential observations of bipedalism, even as a posture, did not occur.

The percentage of time spent as locomotion and using arboreal or terrestrial substrate was calculated as the percentage of 2-min observations that individuals engaged in locomotion or was identified per substrate type, respectively. Individual frequencies were compared between vegetation types, and significance in difference was analyzed using paired *t* tests to account for data point interdependence (because the same individuals are represented in each vegetation type) as data were normally distributed. Because of the smaller sample size, bipedal data were not always normally distributed, and therefore, a Wilcoxon ranked sum was used to investigate differences in bipedal behavior between vegetation type and activity. Although not presented in the main body of this paper, we ran tests considering sex as well as habitat differences on main locomotor mode frequencies and substrate use in different vegetation types to address possible sex differences suggested by prior studies (*36*, *40*), for which each adult individual was grouped into their respective sex and vegetation type categories (e.g., female miombo versus female forest). These data were normally distributed and analyzed using a two-way analysis of variance (ANOVA) (table S5). All statistics were conducted on data within Issa, whereas between site comparisons were a nonstatistical comparison of frequencies, because each site was only represented by one data point (site means), making statistical tests nonmeaningful due to problems of power with a small sample.

A multivariate analysis was chosen to investigate synergistic influence of selected variables on time spent in the trees during locomotion in relation to vegetation type. We fitted a GLMM, with a logit response distribution using the “glmer” function from lme4 package in R studio (version 4.0.5) (*71*) to investigate the role of categorical variables (vegetation type, season, activity, and sex) and their interactions on substrate use (arboreal/terrestrial) during locomotion, with chimpanzee identity set as a random intercept (to account for repeated samples of the same individuals and between-subject variation). Fixed variable categories used for GLMM were the following: activity = feeding/traveling; sex = female/male; vegetation = forest/woodland; season = early dry/late dry/wet, with the first category in each variable as the reference. Dry season was split into early and late to even out sampling effort (more samples in dry than wet season) and to capture possible effect of fires clearing undergrowth (grass) in the woodland in the late dry season. Locomotor mode itself was not included in the model because it was strongly correlated to activity and it was decided activity better reflects ecology than locomotor mode and reduced model parameters. Different combinations of explanatory variables and their interactions were run, and their significance was checked using Wald chi-square test (“Anova” function in car package R studio). Nonsignificant parameters were subsequently dropped from the model in a backward stepwise approach, and the Akaike information criterion (AIC) was used to select the model that minimizes information loss when estimating full reality/the most parsimonious (“best”) model (*72*). Model validity was checked using DHARMA package in R studio. The final selected model is shown in table S6, which had the lowest AIC (2283.7) while avoiding complex three-way interactions. All statistical tests were run in R studio version 4.0.5 (*71*). Levels of significance were set at *P* < 0.05. Means are presented in all tables and figures.

## Supplementary Materials

### This PDF file includes:

Figs. S1 and S2 Tables S1 to S7 References
