# Supplementary material for: Wild chimpanzee behavior suggests that a savanna-mosaic habitat did not support the emergence of hominin terrestrial bipedalism
Source: Sci Adv. 2022 Dec 14;8(50):eadd9752. doi: 10.1126/sciadv.add9752 (PMC9750136; doi:10.1126/sciadv.add9752)
Supplement: Supplementary file 1 — Figs. S1 and S2 Tables S1 to S7 References [file sciadv.add9752_sm.pdf]

Science Advances
AAAS

Supplementary Materials for

Wild chimpanzee behavior suggests that a savanna-mosaic habitat did not support the emergence of hominin terrestrial bipedalism

Rhianna C. Drummond-Clarke *et al.*

Corresponding author: Rhianna C. Drummond-Clarke, rcd23@kent.ac.uk; Alex K. Piel, a.piel@ucl.ac.uk

Sci. Adv. **8**, eadd9752 (2022)
DOI: 10.1126/sciadv.add9752

The PDF file includes:

Figs. S1 and S2
Tables S1 to S7
Legends for data S1 to S3
References

Other Supplementary Material for this manuscript includes the following:

Data S1 to S3

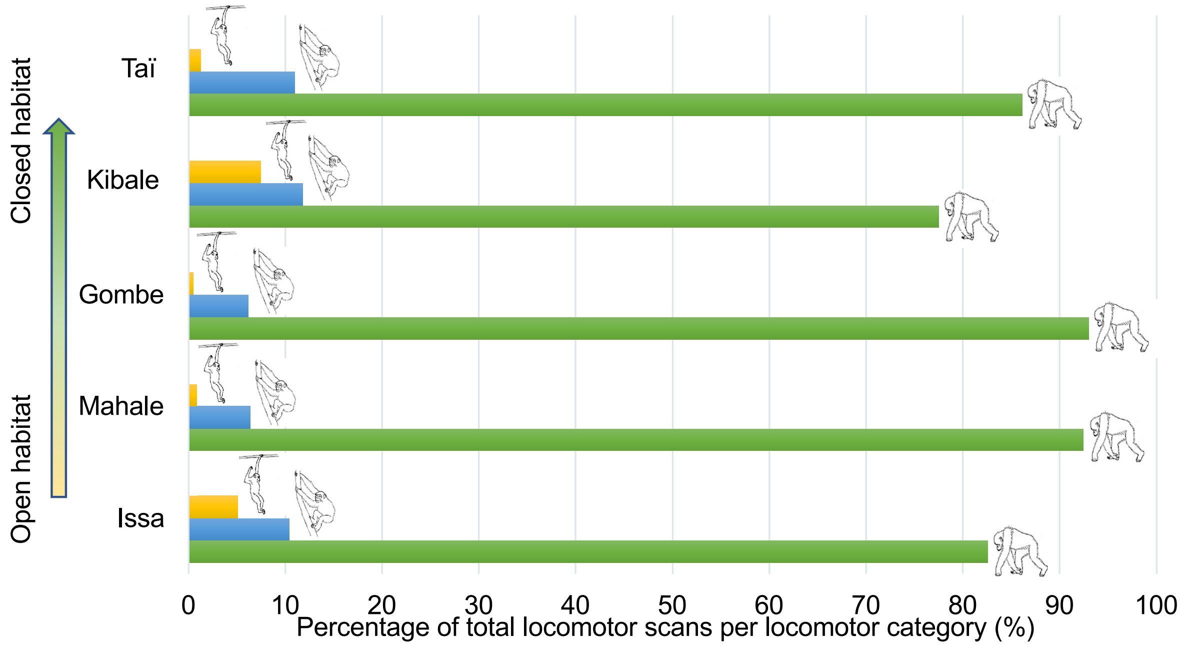

Fig. S1.

Inter-site differences in principle locomotor category frequencies. Bars represent frequency of quadrupedal walking (green), climbing (blue), and suspension (yellow) (see **Table S1** for category definitions). The amount of time spent walking quadrupedally correlates negatively with time spent in the trees. Note that Issa chimpanzees spend less time in quadrupedal walking, and more time engaging in solely- or primarily- arboreal behaviors (i.e. suspension and climbing) than all sites except Kibale. Additionally, Issa chimpanzees spend a surprisingly high proportion of locomotion in suspensory behaviors, a terminal branch behavior that is associated with bipedalism as a safe way to move in thin branches (*41*, *69*) (see also **Fig. S2**). Locomotor categories 'Bipedal' and 'Other' are here not included due to being very rare behaviors (<2% across sites). Mahale, Gombe, and Taï data from (*36*), Kibale data from (*37*). Schematics sourced from (*73*).

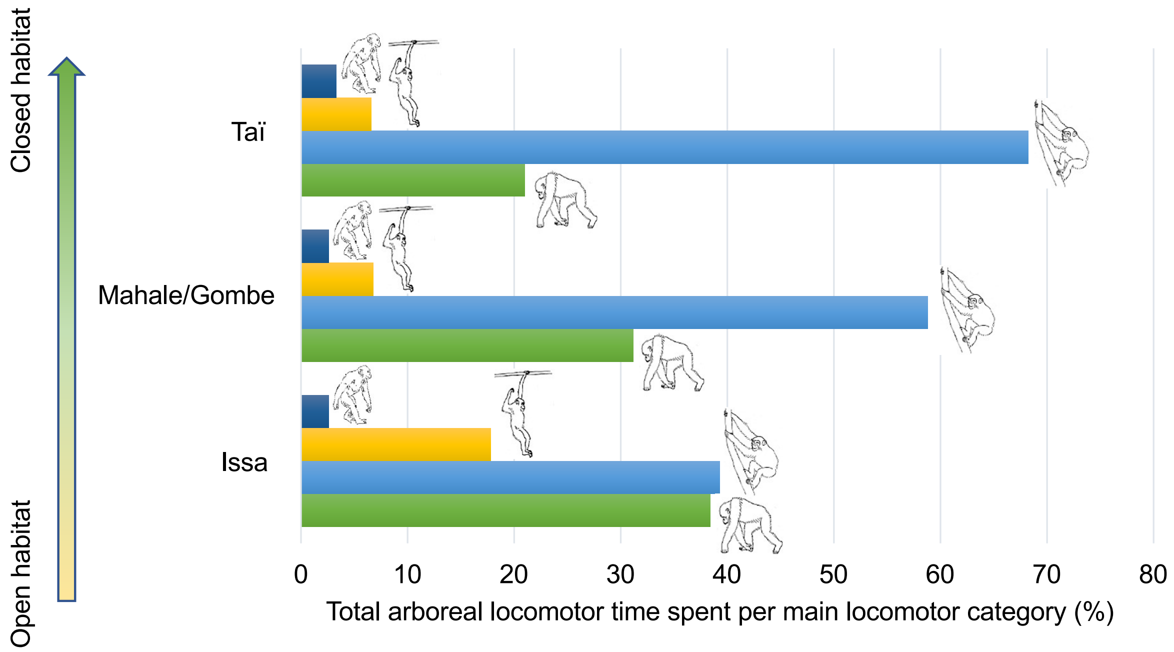

Fig. S2.

Inter-site differences in arboreal locomotor category frequencies. Bars represent frequency of quadrupedal walking (green), climbing (light blue), suspension (yellow), and bipedalism (dark blue) (see **Table S1** for category definitions). When in the trees, Issa chimpanzees engaged in more suspensory locomotion and climbed less, but used a similar amount of bipedalism compared to other chimpanzee communities with available arboreal locomotor data (sourced from (*36*), Taï values estimated from male and female averages). Arboreal locomotor frequencies are not available from Kibale. Once in the trees, there was no significant difference in locomotor behavior between the forest and woodland vegetation types or sexes at Issa, considering main locomotor modes. 'Other' category was not included here due to being a rare behavior (<2% across sites). Schematics sourced from (*73*).

Table S1.

Classification of locomotor and postural behavior following refs. (*37*, *68*, *69*). Categories are used as broader groupings to allow meaningful comparison to past studies (*36*).

Locomotor behavior classification

Category	Mode	Definition
Quadrupedal walk	Quadrupedal Walk	Four limbed locomotion on a roughly horizontal support, torso is horizontal. May be inverted (upside down), with limbs in suspension. Includes tripedal walking, crutching, and running.
Bipedal*	Bipedal Walk	Locomotion on a roughly horizontal support, torso is orthograde, hindlimbs provide propulsion, with minimal support from other body parts. Forelimbs may be used for support.
Climb	Vertical climb	Upwards locomotion on 45-90degree support. Typically uses all four limbs.
	Vertical descent	Downwards locomotion on 45-90 degree support. Typically uses all four limbs. Can be head first, rump first and sideways.
	Sway/ ride[ǂ]	Animal moves side to side/overbalances a flexible vertical support to reach an adjacent or lower object/support.
	Bridge[ǂ]	A gap crossing horizontal movement.
Suspension	Torso-Orthograde suspensory	Torso is upright, majority of weight borne by forelimbs. Arm in suspension (elbow and shoulder joints fully extended).
	Drop[ǂ]	Vertical downward movement with a period of free-flight. Uses no propulsion, just release.
Other	Leap	A gap crossing movement propelled by hindlimbs, involves a period of free flight.

Postural behavior classification

Category	Mode	Definition
NS	Sit	Majority of weight is borne by rump; torso is relatively upright.
	Squat	Weight is borne by feet close to buttocks, knees and hips strongly flexed (<90), torso relatively upright.
Bipedal*	Orthograde stand	Standing on hindlimbs only. Torso is roughly vertical hands may be used for balance.
NS	Pronograde stand	Standing on four, three or two limbs that are extended at the knee/elbow joints and under compression. Torso is horizontal/sub horizontal.
	Orthograde forelimb suspend	Majority of weight is borne by one or two forelimbs, grasping an above support. Elbows and shoulders fully extended, torso is orthograde.
	Pronograde suspend	Majority of weight is borne by both forelimbs and hindlimbs grasping a support above the animal. Torso is horizontal.
	Hindlimb suspend	Hanging upside down by the feet/foot, may swing.
	Lie	Majority of weight is borne by the torso, in a horizontal position on a roughly horizontal support.
	Cling	All four or three limbs are flexed and hands and feet grasp to (sub-) vertical support(s). No weight is borne on the ischia. Torso is near orthograde, exception in infants.

*In analyses, bipedal pools postural and locomotor observations, unless indicated otherwise.

ǂIn more recent categorization schemes these modes would be grouped in the 'Other' locomotor category (e.g., (*37*, *69*)), but are here grouped in categories following (*36*) to permit intersite comparison of general locomotor behavioral profiles.

Table S2.

Number of two-minute scans per adult individual, sex category and vegetation type throughout the study period (2020 and 2021 combined). Note that both sexes spent roughly equal amounts of time in both habitats. Total observation hours = 459 (follows were stopped/not included when the focal individual was not visible for >10 scans in the hour follow).

	Vegetation type season									
	**Riparian Forest**				**Miombo Woodland**					
**Focal sex/** ID	Early dry	Late dry	Wet	**Forest total (N)**	Early dry	Late dry	Wet	**Woodland total (N)**	**Total scans (N)**	**Total scans as locomotion (N)**
**Female**	**1241**	**1558**	**1141**	**3940**	**1077**	**1075**	**637**	**2789**	**6729**	**1336**
Ba.	67	310	149	526	55	89	94	238	764	133
Ju.	293	307	265	865	194	146	137	477	1342	244
Ki.	192	251	107	550	235	121	79	435	985	196
Ko.	205	234	210	649	281	286	64	631	1280	301
Ma.	173	197	193	563	74	267	124	465	1028	189
Za.	311	259	217	787	238	166	139	543	1330	273
**Male**	**962**	**1169**	**822**	**2953**	**1859**	**1459**	**743**	**4061**	**7014**	**1511**
Bo.	142	156	26	324	316	225	125	666	990	197
El.	197	214	136	547	247	209	41	497	1044	209
Im.	146	227	244	617	386	232	108	726	1343	296
Kit.	82	14	76	172	124	62	40	226	398	99
Mb.	123	181	84	388	364	214	84	662	1050	214
Sa.	77	213	115	405	140	257	67	464	869	164
Wa.	195	164	141	500	282	260	278	820	1320	332
**Total**	**2203**	**2727**	**1963**	**6893**	**2936**	**2534**	**1380**	**6850**	**13743**	**2847**

Table S3.

Definitions used for other variables collected as part of this study (activity and substrate type). All activity states not in bold font were grouped together as “other” for this study, as they were rare or not involved in locomotor behaviors.

ACTIVITY STATE	Definition
**feeding**	Involves foraging and food-processing with or without using a tool
**traveling**	Movement not associated with a specific activity such as foraging, feeding etc.
grooming	Involves allo-grooming and self-grooming with or without using a tool.
playing	Play behavior, solitary or between two or more individuals. Appears to be for pleasure.
resting	Any period of inactivity when an individual is not traveling, foraging or engaging in social behavior
looking	Involves inspecting/observing objects, individuals or scenes, or looking out, near and/or far, vigilance
displaying	Any behavior directed towards other individuals of an intimidating/threatening manner.
fighting	Aggressive behavior towards another, or multiple, individuals involving physical contact.
mating	Copulation with another individual.
nest building	Preparing a nest in the tree or on the ground with branches and twigs
other behavior	Any solitary, social or other behavior not related to above activities. Please describe in comments.
**SUBSTRATE**	
arboreal	Vegetation weight bearing structure above ground level (>0m): tree trunk, limb, branch, vine, shrub
terrestrial	Ground, rocky features (boulders, outcrops, cliffs), termite mounds.
**VEGETATION TYPE**	
miombo woodland	Grassy understory, dominated by deciduous *Brachystegia* and *Julbernardia* tree species, low canopy connectivity, open vegetation.
riparian forest	Liana dense or open understory, high canopy connectivity, evergreen, diverse tree species, dense vegetation.

Table S4.

Vegetation structure of forest and woodland vegetation types at Issa Valley, following (*67*). Means given where possible (followed by min and max values in brackets). Two values separated by a comma show min and max values. DBH = (trunk) diameter at breast height, MWU = Mann-Whitney U test.

		forest	woodland	post hoc MWU
**trees**				
	**DBH >10cm**			
	n	32 (18, 43)	15 (12, 19)	NS
	height	11.8	8.8	F>W Z=-4.2 p<0.001
	DBH	21.5	21.2	NS*
	crown width	2.1-2.8	1.7-2.2	NS
	crown height	7.6	5.9	F>W Z= -2.8 p=0.005
	Density per m^2*	0.04	0.02	
	**DBH <10cm**			
	n	89	61	NS
**N lianas**	DBH >10cm	1.2	0	NS
**feeding trees**	n	11.5	7.2	NS
**DBH >10cm**	%	36	47	NS
	% in fruit	4	27.9	NS
**canopy**	cover	68.8 (55.4, 78.3)	44.4 (19.4, 57.1)	F>W Z=-2.7 p= 0.004
	connectivity	26, 100	1, 75	
**understory %**	bare land	76, 100	26, 50	
	grass	0	26, 75	
	herbaceous veg	0	0	
**temperature**	day	23.3 (22.3-24.4)	26.6 (23.9-29)	W>F Z = -3 p=0.001
	night	18.5	18.6	NS
**luminosity**	day	3, 767	14, 440	W>F Z= -3.4 p<0.001
	night	3	8	NS
**slope**		flat/slope	flat-slope	-

*From Piel & Stewart, unpublished data.

Table S5.

Positional behavior characteristics of Issa and other habituated chimpanzee communities. Issa forest/woodland columns represent values for these distinctive vegetation types used equally by the Issa chimpanzee community. All values show mean percentage unless stated otherwise. At Issa 2-min locomotor scans N = 2847 (1336 female, 1511 male). Kibale data from (*37*), Taï (*36*, *40*, *74*), Mahale and Gombe (*36*, *41*).

**Site subspecies**	**Issa (*P. t. s*)**			**Kibale (*P. t. s*)**	**Taï (*P. t. v*)**	**Mahale (*P. t. s*)**	**Gombe (*P. t. s*)**
habitat type	Savanna	*Riparian forest*	*Miombo woodland*	Dense forest	Dense forest	Mosaic forest	Mosaic forest
**Time spent arboreally**							
Male	43.8	*47.9*	*39.3*	-	48.9	32.9	37.4
female	67.9	*75.5*	*60.7*	-	64.8	47.8	68.4
**Time spent as locomotion**	**21**	*19*	*25*	17	15	18	18
**Time spent as locomotion arboreally**							
Male	15.6	*26.7*	*11*	-	14.7	8.2	
Female	40.2	*57.6*	*28.2*		18.2	12	
**Percentage of total locomotor observations marked as bipedal**							
**Overall**	**1**	***1.7***	***0.7***	**1.8**	**1.2**	**0.3**	
Male	0.5	*0.57*	*0.5*	-	1.2	0.3	-
Female	1.6	*2.52*	*0.6*	-	1.2	0.2	
**Arboreal**	**2.6**	***3.8***	***1.7***	**-**	**3.3**	**2.6**	

Time spent moving differed significantly between microhabitats ($F = 6.62$, $p = 0.026$), but not sexes ($F = 0.55$ $p = 0.47$), at Issa. Time spent moving in the trees differed significantly between sexes and microhabitat considering postural and locomotor time (2-way ANOVA sex $F = 16.91$, $p = 0.002$ habitat $F = 7.75$, $p = 0.02$), and during locomotion alone (2-way ANOVA sex $F = 29.65$, $p < 0.001$, habitat $F = 40.71$, $p < 0.0001$).
Sex differences and difference between vegetation type were not significant during locomotor bipedalism overall (Friedmans X^2 $p = 0.4$), nor in the trees ($p = 1$).

Table S6.

GLMM results showing significant influence of vegetation type on support use (arboreal or terrestrial) during locomotion. Reference levels = arboreal, forest, feeding, female, early dry season. Variable categories in brackets show category tested against reference level.

	**Estimate**	**Std. Error**	**z value**	***p*-Value**
Terrestriality (AIC = 2283.7 df = 2794)				
(Intercept)	2.83	0.29	9.83	< 0.0001
**Vegetation type (miombo)**	-1.17	0.18	-6.59	< 0.0001
**Activity (traveling)**	-2.95	0.23	-13.12	< 0.0001
**Season (late dry)**	-0.61	0.18	-3.49	0.0005
**Season (wet)**	0.39	0.18	2.19	0.03
Sex (male)	-0.54	0.39	-1.40	0.16
**Vegetation type (miombo) * season (late dry)**	0.51	0.26	1.96	0.05
**Vegetation type (miombo) * season (wet)**	-0.72	0.28	-2.60	0.009
**Activity (traveling) * sex (male)**	-0.93	0.35	-2.68	0.007

Table S7.

Adult bipedal behavior across chimpanzee communities. Site names in bold are those included in **Fig. 4**, with data available or observation hours N > 100. Issa values in brackets were calculated using the number of 2-minute scans not including scans where the positional mode was marked non-visible. Values not in brackets and for other sites were calculated from total number of observation hours (including non-visible scans), or taken from the available literature. Data for Mahale and Gombe sourced from (*5*, *42*), Taï (*74*), Kibale (*37*), Bwindi (*38*).

**Site**	**Observation hours** (N)	**bipedal observations** (N)	**Rate of bipedalism** (instances per hour)	**Frequency of bipedalism** (%)	**Percentage of bipedal observations spent** (%)	
					**terrestrial**	**foraging**
**Issa**	458 (442)	109	0.24	0.79 (0.82)	14	79
Forest	*230 (218)*	*61*	*0.27*	*0.88 (0.93)*		
Woodland	*228 (224)*	*48*	*0.21*	*0.70 (0.71)*		
**Mahale/ Gombe**	571	97	0.17	0.28	39	80
Taï	430	17*	-	0.2*	-	-
Kibale	55	12	0.22	0.73	-	-
**Bwindi**	247	180	0.73	-	0**	-

* Only locomotor bipedalism was recorded in this study, with bipedal stand subsumed with quadrupedal stand in category "stand", so bipedal stand is not included in the number of bipedal observations and frequency is likely largely underestimated.

**likely biased in arboreal observations due to low habituation and ground visibility.

Data S1. (separate file: timelocomoting+terrestrial_Issa _RCDC.csv)
Issa chimpanzee individual means used to calculate time spent locomoting, and amount of locomotor time spent terrestrially, site average and per vegetation type.

Data S2. (separate file: locosubGLMM_RCDC.csv)
Raw data on Issa chimpanzee locomotor terrestriality collected by RCDC and used in GLMM analysis.

Data S3. (separate file: bipedalscans_RCDC22.csv)
Raw data on Issa chimpanzee bipedal behavior used in analyses, collected by RCDC.

REFERENCES AND NOTES

1. P. Andrews, Last common ancestor of apes and humans: Morphology and environment. *Folia Primatol.* **91**, 122–148 (2019).

2. T. E. Cerling, J. G. Wynn, S. A. Andanje, M. I. Bird, D. K. Korir, N. E. Levin, W. Mace, A. N. MacHaria, J. Quade, C. H. Remien, Woody cover and hominin environments in the past 6 million years. *Nature* **476**, 51–56 (2011).

3. R. Bonnefille, Cenozoic vegetation, climate changes and hominid evolution in tropical Africa. *Glob. Planet Change* **72**, 390–411 (2010).

4. M. Domínguez-Rodrigo, Is the "Savanna Hypothesis" a dead concept for explaining the emergence of the earliest hominins? *Curr. Anthropol.* **55**, 59–81 (2014).

5. K. D. Hunt, The evolution of human bipedality: Ecology and functional morphology. *J. Hum. Evol.* **26**, 183–202 (1994).

6. M. D. Sockol, D. A. Raichlen, H. Pontzer, Chimpanzee locomotor energetics and the origin of human bipedalism. *Proc. Natl. Acad. Sci. U.S.A.* **24**, 12265–12269 (2007).

7. J. Napier, The antiquity of human walking. *Sci. Am.* **216**, 56–66 (1967).

8. K. D. Hunt, The postural feeding hypothesis: An ecological model for the evolution of bipedalism. *S. Afr. J. Sci.* **92**, 77–90 (1996).

9. B. Senut, M. Pickford, D. Gommery, P. Mein, K. Cheboi, Y. Coppens, First hominid from the Miocene (Lukeino Formation, Kenya). *C. R. Geosci.* **332**, 137–144 (2001).

10. T. D. White, B. Asfaw, Y. Beyene, Y. Haile-Selassie, C. O. Lovejoy, G. Suwa, G. Woldegabriel, *Ardipithecus ramidus* and the paleobiology of early hominids. *Science* **326**, 75–86 (2009).

11. N. E. Levin, S. W. Simpson, J. Quade, T. E. Cerling, S. R. Frost, Herbivore enamel carbon isotopic composition and the environmental context of *Ardipithecus* at Gona, Ethiopia. *Geol. Soc. Am. Spec. Paper* **446**, 215–233 (2008).

12. D. F. Su, Y. Haile-Selassie, Mosaic habitats at Woranso-Mille (Ethiopia) during the Pliocene and implications for *Australopithecus* paleoecology and taxonomic diversity. *J. Hum. Evol.* **163**, 103076 (2022).

13. M. J. Schoeninger, H. Reeser, K. Hallin, Paleoenvironment of *Australopithecus anamensis* at Allia Bay, East Turkana, Kenya: Evidence from mammalian herbivore enamel stable isotopes. *J. Anthropol. Archaeol.* **22**, 200–207 (2003).

14. S. Lindshield, R. A. Hernandez-Aguilar, A. H. Korstjens, L. F. Marchant, V. Narat, P. I. Ndiaye, H. Ogawa, A. K. Piel, J. D. Pruetz, F. A. Stewart, K. L. van Leeuwen, E. G. Wessling, M. Yoshikawa, Chimpanzees (*Pan troglodytes*) in savanna landscapes. *Evol. Anthropol.* **30**, 399–420 (2021).

15. B. Senut, M. Pickford, D. Gommery, L. Ségalen, Palaeoenvironments and the origin of hominid bipedalism. *Hist. Biol.* **30**, 284–296 (2018).

16. P. Rodman, H. Mchenry, Bioenergetics and the origin of hominid bipedalism. *Am. J. Phys. Anthropol.* **52**, 103–106 (1980).

17. W. R. Leonard, M. L. Robertson, Energetic efficiency of human bipedality. *Am. J. Phys. Anthropol.* **97**, 335–338 (1995).

18. C. V. Ward, Interpreting the posture and locomotion of *Australopithecus afarensis*: Where do we stand? *Yearb. Phys. Anthropol.* **45**, 185–215 (2002).

19. G. Daver, F. Guy, H. T. Mackaye, A. Likius, J.-R. Boisserie, A. Moussa, L. Pallas, P. Vignaud, N. D. Clarisse, Postcranial evidence of late Miocene hominin bipedalism in Chad. *Nature* **609**, 94–100 (2022).

20. T. C. Prang, K. Ramirez, M. Grabowski, S. A. Williams, *Ardipithecus* hand provides evidence that humans and chimpanzees evolved from an ancestor with suspensory adaptations. *Sci. Adv.* **7**, eabf2474 (2021).

21. D. J. Green, Z. Alemseged, *Australopithecus afarensis* scapular ontogeny, function, and the role of climbing in human evolution. *Science* **338**, 514–517 (2012).

22. S. E. Churchill, T. W. Holliday, K. J. Carlson, T. Jashashvili, M. E. Macias, S. Mathews, T. L. Sparling, P. Schmid, D. J. de Ruiter, L. R. Berger, The upper limb of *Australopithecus sediba*. *Science* **340**, 1233477 (2013).

23. L. R. Berger, J. Hawks, D. J. de Ruiter, S. E. Churchill, P. Schmid, L. K. Delezene, T. L. Kivell, H. M. Garvin, S. A. Williams, J. M. DeSilva, M. M. Skinner, C. M. Musiba, N. Cameron, T. W. Holliday, W. Harcourt-Smith, R. R. Ackermann, M. Bastir, B. Bogin, D. Bolter, J. Brophy, Z. D. Cofran, K. A. Congdon, A. S. Deane, M. Dembo, M. Drapeau, M. C. Elliott, E. M. Feuerriegel, D. Garcia-Martinez, D. J. Green, A. Gurtov, J. D. Irish, A. Kruger, M. F. Laird, D. Marchi, M. R. Meyer, S. Nalla, E. W. Negash, C. M. Orr, D. Radovcic, L. Schroeder, J. E. Scott, Z. Throckmorton, M. W. Tocheri, C. VanSickle, C. S. Walker, P. Wei, B. Zipfel, *Homo naledi*, a new species of the genus *Homo* from the Dinaledi Chamber, South Africa. *eLife* **4**, 09560 (2015).

24. S. G. Larson, W. L. Jungers, M. W. Tocheri, C. M. Orr, M. J. Morwood, T. Sutikna, R. D. Awe, T. Djubiantono, Descriptions of the upper limb skeleton of *Homo floresiensis*. *J. Hum. Evol.* **57**, 555–570 (2009).

25. S. V. Nelson, M. I. Hamilton, Evolution of the human dietary niche: Initial transitions, in *Chimpanzees and Human Evolution*, M. N. Muller, R. W. Wrangham, D. R. Pilbeam, Eds. (HUP, 2017), pp. 286–310.

26. A. G. Henry, P. S. Ungar, B. H. Passey, M. Sponheimer, L. Rossouw, M. Bamford, P. Sandberg, D. J. de Ruiter, L. Berger, The diet of *Australopithecus sediba*. *Nature* **487**, 90–93 (2012).

27. P. S. Ungar, M. Sponheimer, The diets of early hominins. *Science* **334**, 190–193 (2011).

28. C. J. Jolly, The seed-eaters: A new model of hominid differentiation based on a baboon analogy. *Man* **5**, 5–26 (1970).

29. M. D. Rose, Bipedal behavior of olive baboons (*Papio anubis*) and its relevance to an understanding of the evolution of human bipedalism. *Am. J. Phys. Anthropol.* **44**, 247–261 (1976).

30. S. K. S. Thorpe, R. L. Holder, R. H. Crompton, Origin of human bipedalism as an adaptation for locomotion on flexible branches. *Science* **316**, 1328–1331 (2007).

31. J. Moore, Savanna chimpanzees, referential models and the last common ancestor, in *Great Ape Societies*, W. C. McGrew, L. F. Marchant, T. Nishida, Eds. (CUP, 1996), pp. 275–292.

32. C. B. Stanford, Chimpanzees and the behavior of *Ardipithecus ramidus*. *Annu. Rev. Anthropol.* **41**, 139–149 (2012).

33. D. R. Pilbeam, D. E. Lieberman, Reconstructing the last common ancestor of chimpanzees and humans, in *Chimpanzees and Human Evolution*, M. N. Muller, R. W. Wrangham, D. R. Pilbeam, Eds. (HUP, 2017), pp. 22–142.

34. T. D. White, C. O. Lovejoy, B. Asfaw, J. P. Carlson, G. Suwa, Neither chimpanzee nor human, *Ardipithecus* reveals the surprising ancestry of both. *Proc. Natl. Acad. Sci. U.S.A.* **112**, 4877–4884 (2015).

35. K. L. van Leeuwen, R. A. Hill, A. H. Korstjens, Classifying chimpanzee (*Pan troglodytes*) landscapes across large-scale environmental gradients in Africa. *Int. J. Primatol.* **41**, 800–821 (2020).

36. D. M. Doran, K. D. Hunt, Comparative locomotor behavior of chimpanzees and bonobos, in *Chimpanzee Cultures*, R. W. Wrangham, W. C. McGrew, F. de Vaal, Eds. (HUP, 1994), pp. 93–108.

37. L. A. Sarringhaus, L. M. MacLatchy, J. C. Mitani, Locomotor and postural development of wild chimpanzees. *J. Hum. Evol.* **66**, 29–38 (2014).

38. C. B. Stanford, Arboreal bipedalism in wild chimpanzees: Implications for the evolution of hominid posture and locomotion. *Am. J. Phys. Anthropol.* **129**, 225–231 (2006).

39. C. Giuliano, F. A. Stewart, A. K. Piel, Chimpanzee (*Pan troglodytes schweinfurthii*) grouping patterns in an open and dry savanna landscape, Issa Valley, western Tanzania. *J. Hum. Evol.* **163**, 103137 (2022).

40. D. M. Doran, Sex differences in adult chimpanzee positional behavior: The influence of body size on locomotion and posture. *Am. J. Phys. Anthropol.* **91**, 99–115 (1993).

41. K. D. Hunt, Positional behavior of *Pan troglodytes* in the Mahale Mountains and Gombe Stream National Parks, Tanzania. *Am. J. Phys. Anthropol.* **87**, 83–105 (1992).

42. K. D. Hunt, Ecological morphology of Australopithecus afarensis, in *Primate Locomotion: Recent Advances*, E. Strasser, J. Fleagle, A. Rosenberger, H. McHenry, Eds. (Plenum, 1998), pp. 397–418.

43. K. L. Manduell, M. E. Harrison, S. K. S. Thorpe, Forest structure and support availability influence orangutan locomotion in Sumatra and Borneo. *Am. J. Primatol.* **74**, 1128–1142 (2012).

44. S. D. Ban, E. Normand, Spatial cognitive abilities in foraging chimpanzees, in *The Chimpanzees of the Taï Forest: 40 Years of Research*, C. Boesch, R. Wittig, C. Crockford, L. Vigilant, T. Deschner, F. Leendertz, Eds. (CUP, 2019), pp. 440–450.

45. K. R. L. Janmaat, S. D. Ban, C. Boesch, Chimpanzees use long-term spatial memory to monitor large fruit trees and remember feeding experiences across seasons. *Anim. Behav.* **86**, 1183–1205 (2013).

46. K. R. L. Janmaat, Temporal cognition in Taï chimpanzees, in *The Chimpanzees of the Taï Forest: 40 Years of Research*, C. Boesch, R. Wittig, C. Crockford, L. Vigilant, T. Deschner, F. Leendertz, Eds. (CUP, 2019), pp. 451–466.

47. A. K. Piel, P. Strampelli, E. Greathead, R. A. Hernandez-Aguilar, J. Moore, F. A. Stewart, The diet of open-habitat chimpanzees (*Pan troglodytes schweinfurthii*) in the Issa valley, western Tanzania. *J. Hum. Evol.* **112**, 57–69 (2017).

48. H. Pontzer, R. W. Wrangham, Climbing and the daily energy cost of locomotion in wild chimpanzees: Implications for hominoid locomotor evolution. *J. Hum. Evol.* **46**, 315–333 (2004).

49. E. McLester, K. Sweeney, F. A. Stewart, A. K. Piel, Leopard (*Panthera pardus*) predation on a red-tailed monkey (*Cercopithecus ascanius*) in the Issa Valley, western Tanzania. *Primates* **60**, 15–19 (2019).

50. C. Fryns, G. Badihi, A.-S. Crunchant, R. C. Drummond-Clarke, C. Howell, F. Stewart, A. Piel, Interactions between chimpanzees (*Pan troglodytes schweinfurthii*) and cattle (*Bos taurus*) in the Issa Valley, Western Tanzania. *Afr. Primates* **15**, 19–30 (2021).

51. C. M. Monteza-Moreno, M. C. Crofoot, M. N. Grote, P. A. Jansen, Increased terrestriality in a Neotropical primate living on islands with reduced predation risk. *J. Hum. Evol.* **143,** 102768 (2020).

52. S. C. Reynolds, G. N. Bailey, G. C. P. King, Landscapes and their relation to hominin habitats: Case studies from *Australopithecus* sites in eastern and southern Africa. *J. Hum. Evol.* **60**, 281–298 (2011).

53. R. H. Crompton, W. I. Sellers, S. K. S. Thorpe, Arboreality, terrestriality and bipedalism. *Philos. Trans. R. Soc. Lond. B Biol.* **365**, 3301–3314 (2010).

54. A. van Casteren, V. M. Oelze, S. Angedakin, A. K. Kalan, M. Kambi, C. Boesch, H. S. Kühl, K. E. Langergraber, A. K. Piel, F. A. Stewart, K. Kupczik, Food mechanical properties and isotopic signatures in forest versus savannah dwelling eastern chimpanzees. *Commun. Biol.* **1**, 109 (2018).

55. V. Foerster, A. Asrat, C. Bronk Ramsey, E. T. Brown, M. S. Chapot, A. Deino, W. Duesing, M. Grove, A. Hahn, A. Junginger, S. Kaboth-Bahr, C. S. Lane, S. Opitz, A. Noren, H. M. Roberts, M. Stockhecke, R. Tiedemann, C. M. Vidal, R. Vogelsang, A. S. Cohen, H. F. Lamb, F. Schaebitz, M. H. Trauth, Pleistocene climate variability in eastern Africa influenced hominin evolution. *Nat. Geosci.* **15**, 805–811 (2022).

56. C. B. Ruff, Climate and body shape in hominid evolution. *J. Hum. Evol.* **21**, 81–105 (1991).

57. P. E. Wheeler, The thermoregulatory advantages of hominid bipedalism in open equatorial environments: The contribution of increased convective heat loss and cutaneous evaporative cooling. *J. Hum. Evol.* **21**, 107–115 (1991).

58. D. M. Bramble, D. E. Lieberman, Endurance running and the evolution of *Homo*. *Nature* **432**, 345–352 (2004).

59. W. I. Sellers, G. M. Cain, W. Wang, R. H. Crompton, Stride lengths, speed and energy costs in walking of *Australopithecus afarensis*: Using evolutionary robotics to predict locomotion of early human ancestors. *J. R. Soc. Interface* **2**, 431–441 (2005).

60. J. M. DeSilva, K. G. Holt, S. E. Churchill, K. J. Carlson, C. S. Walker, B. Zipfel, L. R. Berger, The lower limb and mechanics of walking in *Australopithecus sediba*. *Science*, **340**, 1232999 (2013).

61. N. E. Thompson, B. Demes, M. C. O'Neill, N. B. Holowka, S. G. Larson, Surprising trunk rotational capabilities in chimpanzees and implications for bipedal walking proficiency in early hominins. *Nat. Commun.* **6**, 9416 (2015).

62. L. Georgiou, C. J. Dunmore, A. Bardo, L. T. Buck, J.-J. Hublin, D. H. Pahr, D. Stratford, A. Synek, T. L. Kivell, M. M. Skinner, Evidence for habitual climbing in a Pleistocene hominin in South Africa. *Proc. Natl. Acad. Sci. U.S.A.* **117**, 8416–8423 (2020).

63. C. J. Dunmore, M. M. Skinner, A. Bardo, L. R. Berger, J. J. Hublin, D. H. Pahr, A. Rosas, N. B. Stephens, T. L. Kivell, The position of *Australopithecus sediba* within fossil hominin hand use diversity. *Nat. Ecol. Evol.* **4**, 911–918 (2020).

64. M. Cazenave, A. Oettlé, T. R. Pickering, J. L. Heaton, M. Nakatsukasa, J. Francis Thackeray, J. Hoffman, R. Macchiarelli, Trabecular organization of the proximal femur in *Paranthropus robustus*: Implications for the assessment of its hip joint loading conditions. *J. Hum. Evol.* **153,** 102964 (2021).

65. S. A. Blumenthal, N. E. Levin, F. H. Brown, J. P. Brugal, K. L. Chritz, J. M. Harris, G. E. Jehle, T. E. Cerling, J. O'Connell, Aridity and hominin environments. *Proc. Natl. Acad. Sci. U.S.A.* **114**, 7331–7336 (2017).

66. J. Altmann, Observational study of behavior: Sampling methods. *Behaviour* **49**, 227–267 (1974).

67. K. L. van Leeuwen, "Landscapes of the apes: Modelling landscape use of chimpanzees and early hominins across an environmental gradient," thesis, Bournemouth University, UK (2019).

68. K. D. Hunt, J. G. H. Cant, D. L. Gebo, M. D. Rose, S. E. Walker, D. Youlatos, Standardized descriptions of primate locomotor and postural modes. *Primates* **37**, 363–387 (1996).

69. S. K. S. Thorpe, R. H. Crompton, Orangutan positional behavior and the nature of arboreal locomotion in hominoidea. *Am. J. Phys. Anthropol.* **131**, 384–401 (2006).

70. K. L. Manduell, H. C. Morrogh-Bernard, S. K. S. Thorpe, Locomotor behavior of wild orangutans (*pongo pygmaeus wurmbii*) in disturbed peat swamp forest, Sabangau, Central Kalimantan, Indonesia. *Am. J. Phys. Anthropol.* **145**, 348–359 (2011).

71. R Core Team, *R: A Language and Environment for Statistical Computing* (R Foundation for Statistical Computing, 2021).

72. K. P. Burnham, D. R. Anderson, K. P. Huyvaert, AIC model selection and multimodel inference in behavioral ecology: Some background, observations, and comparisons. *Behav. Ecol. Sociobiol.* **65**, 23–35 (2011).

73. E. L. R. Saunders, A. M. Roberts, S. K. S. Thorpe, "Positional Behaviour" in *The International Encyclopedia of Primatology*, A. Fuentes, Ed. (Wiley, 2018).

74. D. M. Doran, "Chimpanzee and pygmy chimpanzee positional behaviour: The influence of environment, body size, morphology, and ontogeny on locomotion and posture," thesis, Stony Brook University, NY (1989).
